# SGLT2 inhibitors and NLRP3 inflammasome: potential target in diabetic kidney disease

**DOI:** 10.1590/2175-8239-JBN-2023-0187en

**Published:** 2024-09-09

**Authors:** Paulo André Bispo Machado, André Lass, Bruna Isadora Pilger, Raphaella Fornazari, Thyago Proença de Moraes, Ricardo Aurino Pinho

**Affiliations:** 1 Pontifícia Universidade Católica do Paraná, Laboratório de Bioquímica do Exercício em Saúde, Curitiba, PR, Brazil.; 2 Pontificia Universidade Católica do Paraná, Pós-graduação em Ciências da Saúde, Curitiba, PR, Brazil.; 3 Pontifícia Universidade Católica do Paraná, Curitiba, PR, Brazil.

**Keywords:** Diabetic Nephropathies, Inflammatory Response, NLR Family, Pyrin Domain-Containing 3 Protein, Inflammasome, Sodium-Glucose Transporter-2 Inhibitors, Renal Insufficiency, Chronic

## Abstract

Diabetic kidney disease (DKD) remains the leading cause of chronic kidney disease (CKD) worldwide. The pathogenesis of DKD is influenced by functional, histopathological, and immune mechanisms, including NLRP3 inflammasome activity and oxidative stress. The sodium-glucose cotransporter 2 inhibitors (SGLT2i) have shown metabolic benefits and the ability to slow the progression of DKD in several clinical studies over the years. Recent studies suggest that the antidiabetic activity also extends to inhibition of the inflammatory response, including modulation of the NLRP3 inflammasome, reduction of pro-inflammatory markers and reduction of oxidative stress. Here we review the efficacy of SGLT2i in the treatment of CKD and discuss the role of the inflammatory response in the development of DKD, including its relationship to the NLRP3 inflammasome and oxidative stress.

## Clinical Summary

- Diabetic kidney disease is the leading cause of chronic kidney disease
- The NLRP3 inflammasome is a key regulator of the inflammatory response in the development of diabetic kidney disease
- SGLT2 inhibitors have shown the ability to slow diabetic kidney disease in several clinical trials
- SGLT2 inhibitors may slow the progression of diabetic kidney disease by inhibiting the NLRP3 inflammasome

## Chronic Kidney Disease: Definition and Epidemiology

Chronic kidney disease (CKD) is a public health problem with global impact, characterized by a progressive and irreversible loss of kidney function^1^. In recent years, an increase in the incidence of CKD has been estimated as a result of demographic change, with CKD affecting approximately 9% of the world’s population^2^. Crucial to its characterization is the time requirement, defined by the presence of damage (albuminuria ≥ 30 mg/day) and/or a glomerular filtration rate < 60 mL/min/1.73 m^2^ for a period greater than or equal to 3 months with progressive, irreversible, and usually asymptomatic loss of kidney function^3^.

CKD has a multifactorial etiology and its main causes are diabetes mellitus (DM), systemic arterial hypertension (SAH), obesity and a family history^2,3^. DM is an intermediate metabolic disorder characterized by persistent elevations of plasma glucose levels. The prevalence of DM has increased significantly in recent years, mainly due to sedentary lifestyles, obesity, and increased life expectancy. In 1985, about 30 million cases were reported worldwide, rising to 415 million in 2015 and an estimated 642 million cases by 2040^4^.

The development of diabetic kidney disease (DKD) is mainly related to diabetes lifespan and the strictness of glycemic control, with several macrovascular and microvascular associated complications, including peripheral arterial occlusive disease, diabetic retinopathy and DKD or diabetic nephropathy (DN) – the most common chronic complications of DM. It is estimated that approximately 40% of individuals with DM develop CKD, which manifests as DKD. This makes it the leading cause of CKD worldwide3 and a major cause of death in these patients^5^.

The pathogenesis of DKD is influenced by functional, histopathological, and immune mechanisms. From a functional point of view, chronic hyperglycemia causes hyperfiltration and an increase in glomerular pressure, which is clinically characterized by an imbalance in the muscle tone of the afferent and efferent arterioles, resulting in an increase in glomerular filtration rate (GFR), glomerular hypertension, and increased excretion of albumin in the urine to levels greater than 30 mg/g^3^. Histologically, this is manifested by nodular and/or diffuse glomerulosclerosis, tubulointerstitial fibrosis, mesangial dilatation and basement membrane thickening, all changes that are considered characteristic of DKD^6^.

Over time, this sustained increase in GFR associated with glomerular hypertension leads to mechanical stress on the filtration barrier, resulting in loss of nephron function, which in turn leads to proteinuria and the development of CKD^7^. One cohort (RIACE) has shown that glomerular hyperfiltration is an independent factor for death from any cause in patients with type 2 DM (T2DM)^8^. Minutolo et al.^9^ showed that the presence of albuminuria is associated with increased mortality and cardiovascular events in patients with DKD compared with patients with CKD of non-diabetic etiology, with the risk directly related to the severity of albuminuria.

## Pharmacological Treatment

Pharmacological treatment is an important pillar in the treatment of DN, acting mainly on glycemic control. In the early stages of both type 1 DM (T1DM) and T2DM, microvascular and macrovascular complications can be reduced by strict glycemic control^10^.

Oral diabetes medications can work in a variety of ways, including lowering hepatic glucose production (biguanides/metformin), increasing insulin secretion (glinides and sulphonylureas), lowering intestinal glucose absorption (alpha-glucosidase inhibitors), increasing peripheral insulin sensitivity (thioglitazones), lowering gastric emptying (GLP1 agonists), increasing GLP1 half-life (DPP4 inhibitors) and, more recently, stimulating of urinary glucose excretion (SGLT2 inhibitors)^11^.

Angiotensin II converting enzyme (ACE) inhibitors or aldosterone receptor blockers (ARBs) are also key drugs in the treatment of DKD, as they can reduce hyperfiltration and glomerular hypertension by vasoconstriction of the afferent arteriole and vasodilation of the efferent arteriole. Important clinical studies have shown that the use of ACE inhibitors in patients with albuminuria reduces the progression of CKD. These drugs are responsible for the neurohormonal control of the kidneys and their use allows the optimization of kidney hemodynamics due to the pro-inflammatory state present in kidney patients^12,13^. Despite this improvement, these drugs are not able to eliminate the long-term increased risk of mortality in these patients^14,15^.

## SGLT2 Inhibitors

Sodium-glucose cotransporter 2 inhibitors (SGLT2i), a new class of oral antidiabetic agents launched in 2012 to slow disease progression by lowering blood glucose levels via the urine^16^, have shown metabolic benefits and the ability to slow DKD progression in several clinical studies in recent years.

SGLT2i act on the proximal kidney tubules and inhibit glucose reabsorption independently of insulin or pancreatic beta cell function. In this sense, SGLT2i has been the subject of large studies due to their proven benefit on cardiovascular and kidney outcomes^17^, mainly because they provide improved glycemic control by regulating glycosylated hemoglobin, fasting glucose and postprandial glycemia, blood pressure reduction due to urinary sodium loss^18^, and weight loss^19^.

In the DAPA-CKD trial, dapagliflozin use significantly reduced the risk of kidney function loss (estimated by GFR), CKD progression and kidney-related death in patients with CKD, regardless of blood glucose status^20^. The study also showed that non-diabetic patients with CKD who received dapagliflozin had improved kidney function (also estimated by GFR) and a lower risk of kidney-related death. Another landmark study in the medical literature is the EMPA Kidney trial, which showed that empagliflozin reduced the risk of kidney disease progression or cardiovascular death in patients with CKD by 28% compared to placebo^21^.

SGLTs are ATP-dependent transmembrane transporters that act as carriers of glucose, ions and amino acids, with the most studied subtypes being SGLT1 and SGLT2^22^. The SGLT1 subtype is less specific as it is found in various organs such as the small intestine, brain, heart and distal segments of the proximal kidney tubules (segment S3) and is responsible for the reabsorption of approximately 10% of the glucose filtered by the kidney^17^. Because they are located in different parts of the body, SGLT1 subtype inhibitors can cause various adverse effects, such as gastrointestinal disorders, which led to the discontinuation of studies that initially involved this subtype^22^. On the other hand, the SGLT2 subtype is a specific transporter of kidney tissue with low affinity and high glucose reabsorption capacity, which is found in the apical membrane of the proximal cells of the kidney tubules (segment S1) and is responsible for the reabsorption of approximately 90% of the glucose filtered by the kidney^16^.

The first drug of the SGLT2i class to be discovered was phlorizin, a molecule found in the leaves and buds of apple trees^23^. However, due to its low selectivity for SGLT2 and various side effects, mainly gastrointestinal, the drug ceased to be used^24^. Nevertheless, other drugs have been developed based on its molecular structure that increase selectivity for SGLT2, thereby reducing side effects and increasing bioavailability.

To date, there are approximately 9 molecules that can inhibit the SGLT2 transporter, but only 4 have been approved by the Food and Drug Administration (FDA) for the treatment of DM2, namely: empagliflozin, dapagliflozin, canagliflozin and ertagliflozin^25^. Dapagliflozin was the first drug in this class to be approved in 2012, while empagliflozin was approved in 2014^26^. In the class, empagliflozin has the highest affinity for SGLT2 compared to SGLT1^27,28,29^.

In the proximal tubules, the sodium-to-glucose ratio is 1:1 for SGLT2 (S1 segment) and 2:1 for SGLT1 (S3 segment)^30^. The reabsorbed glucose is then transferred to the interstitium by facilitated diffusion through glucose transporters (GLUTs) present in the basolateral membrane of the cell^31^.

It is estimated that a healthy adult can filter approximately 180 g of glucose per day^32^. At higher levels, the ability of the kidney to reabsorb glucose is impaired, so that elevated glucose levels are detected in urine tests, causing symptoms of non-glycemic control such as polyuria and polydipsia. Paradoxically, this loss of glucose in diabetics leads to hypertrophy of the cells of the proximal kidney tubules, where the SGLT2 transporter is located, with an increase in its expression in the apical membrane, increasing the capacity for reabsorption of kidney glucose by this transporter to preserve energy in the body.

In this way, there is an increase in the reabsorption of glucose and sodium, which leads to a decrease in the amount of NaCl in the lumen of the distal tubules near the dense macula. This initiates tubuloglomerular feedback, which relaxes the tone of the kidney afferent arteriole, increasing blood flow and GFR, causing glomerular hypertension^32^. This process further increases the already high glycemic levels, leading to increased insulin secretion and body weight gain^33^.

By blocking the SGLT2 transporter, this class of drugs promotes a reduction in sodium absorption, which has important effects on the dynamics of kidney filtration. The first effect is related to the excretion of sodium in the urine, resulting in a decrease in blood pressure and effective circulating volume^34^. The second effect is related to tubuloglomerular feedback, in which the increased presence of sodium in the macula densa causes vasoconstriction of the afferent arteriole, reducing glomerular hyperfiltration^34^.

Experimental studies in rats have shown that the use of SGLT2i can reduce hyperfiltration by about 25% through the decrease in the reabsorption of NaCl and glucose in the proximal tubules, thus reducing intraglomerular pressure (5–8 mmHg)^35^. The reduction of intraglomerular pressure is an important factor in the reduction of albuminuria caused by SGLT2i^35^. Another hypothesis about the mechanism of action of SGLT2i is related to changes in lipid metabolism and increased production of ketone bodies^36^ as well as hemodynamic changes^35^.

## Inflammatory Responses and Kidney Oxidative Stress

Recent studies have shown that the inflammatory response and oxidative stress are important in the development and progression of DKD. This is mainly due to the innate immune system, the increase in reactive oxygen species (ROS) and the increase in pro-inflammatory cells and cytokines such as interleukin-1ß, interleukin-18, tumor necrosis factor alpha (TNF-α) and nuclear factor kB (NF-kβ)^37,38,39,40^.

Historically, DKD was believed to be a metabolic disease characterized only by hyperglycemia associated with cardio-metabolic risk factors such as high blood pressure, obesity and dyslipidemia^38^. Despite this, a number of studies have highlighted the complexity of the disease and described the importance of innate immunity, inflammatory response and oxidative stress in its development and progression^39,40^.

The inflammasome NLRP3 complex, an intracytoplasmic receptor found in cells of the innate immune system, is a key regulator of this inflammatory mechanism. It is known to play a role in the pathophysiology of neurodegenerative and cardiovascular diseases such as Alzheimer’s and heart failure, and has recently been studied in relation to metabolic diseases such as gout and T2DM^40^.

Additionally, oxidative stress is a crucial factor in the pathogenesis of DKD. In DKD, oxidative damage to the kidneys is caused by an increase in reactive oxygen species (ROS) production and a decrease in antioxidant defense mechanisms. In DKD, hyperglycemia, dyslipidemia and activation of the renin-angiotensin-aldosterone system contribute to oxidative stress^41,42^.

ROS are generated by mitochondrial respiratory chain enzymes and can directly damage lipids, proteins and DNA as well as activate multiple signaling pathways that contribute to inflammation and fibrosis. For example, hyperglycemia activates a specific metabolic pathway involving diacylglycerol (DAG), protein kinase C (PKC), and NADPH oxidase, resulting in the production of ROS^42^.

In DKD, chronic hyperglycemia increases ROS production and induces cell apoptosis, contributing to the progression of diabetic complications. Thus, reducing oxidative stress through various mechanisms (lifestyle modifications, medications and targeted antioxidant therapies) can prevent or delay the progression of DKD^43^.

## The Innate Immunity

Innate immunity is the body’s first line of defense against infectious agents. It consists primarily of natural killer cells, the complement system and the phagocytic system. The phagocytic system, especially represented by macrophages, is important for the production of pro-inflammatory cytokines, ROS and metalloproteinases, which are responsible for local inflammation, atherogenesis, and tissue damage^44-46^.

Several studies have already demonstrated that the magnitude of infiltration by these cells in the kidney parenchyma is directly related to the degree of functional dysfunction of the kidneys, suggesting a possible causal relationship^47,48^. Furuta et al. were among the first to observe this phenomenon in 1993 when studying macrophage infiltration in biopsies from DM patients^49^ and suggested that the phagocytic system may be related to the irreversible damage to the glomerular structure. In 2006, Nguyen et al. demonstrated an accumulation of macrophages and immune cells in the kidney tubules in biopsy tissues from patients with DKD, linking the intensity of interstitial accumulation to the decline in kidney function, demonstrating the importance of these cells in the pathogenesis of the disease^50^.

Ninichuk et al. showed the following year that blocking CCR-1, a chemokine that attracts macrophages to kidney tissue, was able to reduce tubular fibrosis and interstitial inflammation in a rat model of DKD^51^. In 2017, Klessens et al. used a similar method to demonstrate accumulation of macrophages in the glomeruli from biopsy of 88 patients with kidney disease from diabetes and linked phagocytic accumulation as an important factor in disease progression^52^. The increase in expression of chemokines and adhesion molecules (CCR-1, CCR-2) has already been observed in kidney biopsies from patients with diabetic kidney disease^53,54^. Studies evaluating the use of antagonists of these receptors have already demonstrated benefits in animal models, resulting in a reduction in macrophage infiltration in the kidney parenchyma and a decrease in albumin excretion^54^.

## Immune System Recognition Receptors

Cells of the immune system have specific receptors known as pattern recognition receptors (PRR), which are responsible for detecting structural molecules of microorganisms known as pathogen-associated molecular patterns (PAMPs), and toxins or fragments of DNA and RNA resulting from cell damage, such as nucleic acids, intracellular proteins such as HMGB1 (High-mobility group B1), HSPs (heat shock proteins) and messenger RNA, known as tissue damage-associated molecules (DAMPs)^55^.

In the context of DKD, the chronic hyperglycemic state can cause endothelial and cellular lesions that release DAMPs and PAMPs into the interstitium, which are recognized by PRRs. PRRs, which may be found in the plasma membrane or cell cytoplasm, work together to recognize stress signals produced by cells during infection or cell injury^56^. When these receptors are present on the membrane, they are referred to as Toll Like Receptors (TLRs). When expressed on the surface of kidney cells, they are largely responsible for inducing the immune response by recognizing DAMPs and PAMPs^57^. These DAMPS and PAMPS lead to activation of the innate immune response^58^, leading to kidney inflammation and tissue damage mainly through activation of the NF-kB pathway^59^.

C-type lectin receptors (CLRs), retinoic acid-inducible gene-like receptors (RLRs), absence-of-melanoma-like receptors (ALRs), and nucleotide-binding and oligomerization domain (NOD)-like receptors (NLRs) are all examples of PRRs^60,61^. The NLRs are responsible for the host’s second line of defense and are further subdivided into subtypes such as NLRP1, NLRP3, NAIP, and NLRC4^62^. Each has the ability to be activated specifically by certain groups of PAMPs and/or DAMPs, and their activation leads to the formation of cytoplasmic protein complexes known as inflammasomes.

## The NLRP3 Inflammasome

NLRs are cytoplasmic receptors that act as the host’s second line of defense and have three functional structures: a central oligomerization domain (NATCH), a C-terminal domain rich in leucine repeats (LRR), and an N-terminal domain responsible for stimulus transduction with the subdomains CARD (Caspase activation and recruitment domain) or PYD (pyrin domain)^63^. These subdomains are responsible for triggering specific immune responses, causing the NLRs being further subdivided into other subtypes, such as NLRP1, NLRP3, NAIP and NLRC4^62^. The receptor subtype whose mechanism of function is best understood in the context of DKD is NLRP3^64,65^.

In the NLRP3 complex, the N-terminal domain, responsible for stimulus transduction has a PYD subdomain. On the other hand, the pro-caspase-1 enzyme has a CARD subdomain. In order for the interaction between the NLRP3 protein and the pro-caspase-1 enzyme to occur, an adaptation protein (ASC) is therefore required that has the PYD and CARD domains^65^. Through its PYD domain, this protein binds to NLRP3, and on the opposite side, the presence of its CARD subdomain allows the recruitment of caspase-1 (via CARD-CARD interaction). After this interaction, oligomerization of the NLRP3 complex occurs with autocleavage of the caspase-1 enzyme and subsequent activation of IL-1β and IL-18. Furthermore, cell death occurs mediated by gasdermin-D (GSDMD) activation, a process known as pyroptosis^65,66^.

The activation of the NLRP3 inflammasome requires two complementary mechanisms. The first mechanism is related to the stimulation of PRRs located in the cell membrane through PAMPs or DAMPs, which triggers an intracellular signaling cascade that culminates in the activation and translocation of the transcription factor NF-kB to the cell nucleus^67^. When NF-kB enters the nucleus, it stimulates the transcription of pro-IL-1β, pro-IL-18 and the expression of the NLRP3 enzyme^64^.

This is followed by the second activation signal, which is the cleavage of precursors previously activated by NF-kB in the cell nucleus^68,69^. This is triggered by the presence of DAMPs or other stimuli, such as an increase in the concentration of ROS, extracellular ATP, potassium efflux, uric acid crystals and nuclear proteins such as HMGB1^69,70^. These molecules can activate and oligomerize the NLRP3 complex, involving the recruitment and maturation of caspase enzymes, which, when performing a self-cleavage process, activate IL-1β and IL-18, which are released into the extracellular environment via channels created in the cytoplasmic membrane^71,72^ to exert their pro-inflammatory action. In addition, GSDMD is cleaved and its N-terminal subdomain is released, which penetrates the plasma membrane of the cell forming pores. This process, known as pyroptosis, is a form of programmed cell death that generates an intense inflammatory response by allowing cell osmolysis, DNA lysis and the release of cell components and inflammatory mediators such as IL-1β and IL-18^67,68, 69^.

## The Role of the NLRP3 Complex in Diabetic Kidney Disease

Despite being more commonly found in cells of the immune system, reports have already shown that the constituent molecules of inflammasomes are also present in other cells of the body, e.g. podocytes and mesangial cells in the kidney system^72,73^. The NLRP3 subtype is most abundant in the kidneys, and activation of this complex is involved in the development and progression of DKD^72,74^.

The discovery of the NLRP3 complex as an inflammatory component involved in the pathogenesis of DKD has led to the development of therapies aimed at inhibiting its activity to attenuate kidney injury^73,74,75^. In the context of DKD, modulation of the NLRP3 complex has been shown to prevent inflammation and slow the progression of fibrosis^67^. For example, knockout of the NLRP3 enzyme reduced inflammation and kidney fibrosis in diabetic mice^75^ and knockout of the ASC enzyme attenuated proteinuria and glomerular damage in mice on a high-fat diet^76^. Thus, attenuating the inflammatory response by modulating the NLRP3 complex has become a new therapeutic strategy in the context of kidney diseases, especially those related to DM, aiming to reduce disease progression by reducing the inflammatory response mediated by the NLRP3 complex.

One of the most important studies involving the relationship between the NLRP3 complex and DKD was conducted in 2015 by Shahzad et al. The study showed that knockout of the NLPR3 enzyme or caspase-1 in diabetic mice was able to reduce albuminuria and extracellular matrix accumulation in these animals, but without changing glycemic levels or body weight^66^. The same study also showed that circulating levels of IL-1β and IL-18 and kidney expression of NALP3 were elevated in DKD mice and that this increase preceded the process of albuminuria and mesangial expansion, implying that NLRP3 activation could be a trigger for DKD. Additionally, pharmacological inhibition of the IL-1 receptor was able to reduce albuminuria and mesangial expansion in diabetic mice.

Wu et al. demonstrated in 2018 that knocking out the NLRP3 enzyme in diabetic mice also protected against the progression of diabetic kidney disease. This resulted in improved creatinine clearance and urinary albumin/creatinine ratio, as well as histopathological consequences such as improved glomerular hypertrophy and expanded mesangial and interstitial fibrosis compared to the control group^75^.

In the same line, several drugs from the oral antidiabetic class have demonstrated potential to inhibit the inflammatory response and modulate the NLRP3 complex pathway in diabetic patients and in animal models. Examples include sulfonylureas, biguanides, glitazones, the glucagon-like peptide 1 (RA-GLP-1) receptor agonists (albiglutide, dulaglutide and exenatide), DPP4 inhibitors and, more recently, SGTL2 inhibitors^77,78^.

Chronic glycemic exposure of kidney tubule cells causes changes in cellular metabolism, including increased expression of pro-inflammatory cytokines, growth and pro-fibrotic factors and reactive oxygen species. Thus, inhibition of glucose reabsorption by proximal tubule cells may be one mechanism of action by which SGLT2i reduces these adverse effects^79^.

## SGLT2 Inhibitors and the Inflammatory Response

Recent studies have shown that SGLT2 inhibitors may inhibit activation of the NLRP3 inflammasome in several animal models, including obesity, lung injury, myocardial infarction, DKD, depression and atherosclerosis. This inhibition is thought to occur through a number of mechanisms, including reduction of glucose uptake and oxidative stress, and modulation of the gut microbiome. The main articles investigating the possible relationship between SGLTi and the NLRP3 complex and their findings, are shown in Table 1.

**Table 1:** Studies evaluating the possible relationship between SGLT2I and NLRP3 complex

Title/Author	Year	Study design/Model	Aim of the study	Drug/Delivery method	Inflammasome Results
**In vivo studies**					
Empagliflozin Protects against Diet-Induced NLRP-3 Inflammasome Activation and Lipid Accumulation ***Benetti et al.***	2016	Experimental Murine model of diet-induced obesity (Male C57BL/6J mice)	Evaluate the ability of Empagliflozin to affect body weight and NLRP3 inflammasome activation	Empagliflozin Mixed in diet (1 mg/kg, 3 mg/kg or 10 mg/kg)	Decrease in Caspase-1 activation and IL-1β production in mice treated with Empagliflozin (3 mg/kg and 10 mg/kg)
The SGLT-2 Inhibitor Dapagliflozin Has a Therapeutic Effect on Atherosclerosis in Diabetic ApoE^-/-^ Mice ***Leng et al.***	2016	Experimental Diabetic atherosclerosis in diabetic mice (Male ApoE−/− mice and C57BL/6J)	Explore the efficacy of Dapagliflozin on atherosclerosis and the influence on the ROS-NLPR3-Caspase pathway	Dapagliflozin Oral gavage (1 mg/kg)	Serum levels of NLRP3, IL-1β and IL-18 were reduced after 12 weeks of treatment with Dapagliflozin
SGLT-2 Inhibition with Dapagliflozin Reduces the Activation of the Nlrp3/ASC Inflammasome and Attenuates the Development of Diabetic Cardiomyopathy in Mice with Type 2 Diabetes. Further Augmentation of the Effects with Saxagliptin, a DPP4 Inhibitor ***Ye et al.***	2017	Experimental Diabetic cardiomyopathy in mice (Type 2 diabetic (BTBR ob/ob) and wild-type (WT) mice)	To assess if Dapagliflozin could attenuate the myocardial dysfunction and NLRP3 activation in diabetic mice	Dapagliflozin Mixed in diet (1 mg/kg)	Dapagliflozin attenuated the activation of the NLRP3 inflammasome (lower mRNA levels of ASC, NALP3, IL-1β and Caspase-1)
Combined SGLT2 and DPP4 Inhibition Reduces the Activation of the Nlrp3/ASC Inflammasome and Attenuates the Development of Diabetic Nephropathy in Mice with Type 2 Diabetes ***Birnbaum et al.***	2018	Experimental Diabetic Nephropathy in mice (Male BTBR ob/ob and WT mice)	Assessed whether Dapagliflozin attenuates the NLRP3 activation and progression of DN in mice	Dapagliflozin Mixed in diet (1 mg/kg)	Dapagliflozin attenuated the activation of the NLRP3 inflammasome after 8 weeks
The SGLT2 inhibitor dapagliflozin attenuates the activity of ROS-NLRP3 inflammasome axis in steatohepatitis with diabetes mellitus ***Leng et al.***	2019	Experimental Steatohepatitis in diabetic mice (ApoE–/– mice)	Evaluate the effects of Dapagliflozin on liver injury and the influence in oxidative stress and NLRP3 activity	Dapagliflozin Oral gavage (1 mg/kg)	Dapagliflozin reduced the activity of NLRP3 inflammasome in the liver (NLRP3, Caspase-1, IL-1β and IL-18)
Empagliflozin Blunts Worsening Cardiac Dysfunction Associated with Reduced NLRP3 (Nucleotide-Binding Domain-Like Receptor Protein 3) Inflammasome Activation in Heart Failure ***Byrne et al.***	2020	Experimental Mouse model of heart failure (Male C57BL/6J mice and male Dahl salt-sensitive rats)	Evaluate the effects of Empagliflozin on the NLRP3 inflammasome in a mice model of heart failure	Empagliflozin Oral gavage (10 mg/kg)	Empagliflozin reduced the levels of IL-1B and IL-18, the infiltration of macrophages and transcript levels of NLRP3, NF-kB and Caspae-1
Dapagliflozin and Ticagrelor Have Additive Effects on the Attenuation of the Activation of the NLRP3 Inflammasome and the Progression of Diabetic Cardiomyopathy: an AMPK-mTOR Interplay ***Chen et al.***	2020	Experimental Diabetic cardiomyopathy in diabetic mice (BTBR and WT mice)	To evaluate whether Dapagliflozin (and Ticagrelor) would attenuate the progression of diabetic cardiomyopathy in T2DM in mice	Dapagliflozin Mixed in drinking water (1.5 mg/kg)	Dapagliflozin reduced mRNA levels of ASC, Caspase-1 and NLRP3 after 12 weeks
SGLT2 inhibition modulates NLRP3 inflammasome activity via ketones and insulin in diabetes with cardiovascular disease Kim et al.	2020	Randomized Control Trial Adults (20–79 years) with T2DM (Humans)	Investigate the effects of Empagliflozin over the NLRP3 inflammasome in patients with T2DM and high-risk of cardiovascular disease	Empagliflozin Oral – 10 mg or 25mg	Empagliflozin reduced levels of IL-1B in macrophages and mRNA levels of IL-1β , TNF-A and NLPR3 after 30-days
Dapagliflozin, an SGLT2 inhibitor, ameliorates acetic acid-induced colitis in rats by targeting NFκB/AMPK/NLRP3 axis ***El Rous et al.***	2021	Experimental Ulcerative Colitis in rats (Male Sprague–Dawley rats)	Investigate the effects of Dapagliflozin in a rat model of Ulcerative Colitis, and the possible modulation of the NLRP3 complex/NF-kB	Dapagliflozin Oral gavage (5 mg/kg or 10 mg/kg)	Seven days of treatment with Dapagliflozin (10 mg/kg) was able to suppress the signaling of NF-kB as well NLRP3/Caspase-1 activity
Ticagrelor and Dapagliflozin Have Additive Effects in Ameliorating Diabetic Nephropathy in Mice with Type-2 Diabetes Mellitus ***Birnbaum et al.***	2021	Experimental Diabetic Nephropathy in mice (Male BTBR ob/ob mice)	Assessed whether Dapagliflozin (or its association with Ticagrelor) attenuates the NLRP3 activation and progression of DN in mice	Dapagliflozin Mixed in drinking water (1.5 mg/kg)	Dapagliflozin attenuated the activation of the NLRP3 inflammasome after 12 weeks
The SGLT-2 inhibitor empagliflozin improves myocardial strain, reduces cardiac fibrosis and pro-inflammatory cytokines in non-diabetic mice treated with doxorubicin ***Quagliariello et al.***	2021	Experimental In vitro (cardiomyocyte) and In vivo (mouse) (Female C57BL/6 mice)	To evaluate the effects of Empagliflozin (and its association with Doxorubicin) over the NLRP3 inflammasome in cell cultures of cardiomyocytes and in a in vivo model	Empagliflozin Concentrations of 50, 100 and 500 nM (In vitro) and oral gavage (10 mg/kg – In vivo)	Empagliflozin decreased the NF-kB activity (dose-dependent way) and also the activity of the NLRP3 complex
Crosstalk Among NLRP3 Inflammasome, ET_B_R Signaling, and miRNAs in Stress-Induced Depression-Like Behavior: a Modulatory Role for SGLT2 Inhibitors ***Muhammad et al.***	2021	Experimental Depression model in rats (Male Wistar rats)	To evaluate the effects of Dapagliflozin as a therapeutic modality in an animal model of depression, and the possible relationship with the NLRP3 Inflammasome	Dapagliflozin P.O (1 mg/kg)	Dapagliflozin was able to suppress the levels of IL-1β and IL-18 as well the activity of the NLRP3 complex after 4 weeks
SGLT2 inhibitor counteracts NLRP3 inflammasome via tubular metabolite itaconate in fibrosis kidney ***Ke et al.***	2022	Experimental Kidney fibrosis in an ischemia/reperfusion model in mice (Male C57BL/6J mice)	To evaluate whether Dapagliflozin would reduce kidney fibrosis through inhibition of the NLRP3 complex in an ischemia/reperfusion model	Dapagliflozin Oral gavage (1 mg/kg)	Dapagliflozin reversed energy metabolism and inflammation alterations and prevented the NLRP3 activation
The dynamic interplay between AMPK/NFκB signaling and NLRP3 is a new therapeutic target in inflammation: Emerging role of dapagliflozin in overcoming lipopolysaccharide-mediated lung injury ***El-Fattah et al.***	2022	Experimental Acute lung injury in rats (Male Sprague-Dawley rats)	Assessed the role of Dapagliflozin in a model of lipopolysaccharide-induced lung injury in rats	Dapagliflozin Oral gavage (5 mg/kg or 10 mg/kg)	Dapagliflozin mitigated the lung injury, reduced oxidative stress and inhibited NLRP3 inflammasome and caspase-1 activity through AMPK/NFkB regulation
Dapagliflozin Alleviates Renal Fibrosis by Inhibiting RIP1-RIP3-MLKL-Mediated Necroinflammation in Unilateral Ureteral Obstruction ***Xuan et al.***	2022	Experimental Renal fibrosis in a unilateral ureter obstruction model (Male Sprague-Dawley rats)	Investigate whether Dapagliflozin could provide renal protection against renal fibrosis through anti-inflammatory effects in a rat model of ureter obstruction	Dapagliflozin Oral gavage (10 mg/kg)	Dapagliflozin alleviated renal fibrosis, expression of pro-inflammatory parameters (IL-1β, IL-18 and NLRP3) and oxidative stress
Sodium-Glucose Co-transporter-2 Inhibitor of Dapagliflozin Attenuates Myocardial Ischemia/Reperfusion Injury by Limiting NLRP3 Inflammasome Activation and Modulating Autophagy ***Yu et al.***	2022	Experimental Mouse model of myocardial ischemia/reperfusion injury (Male C57BL/6J mice)	To assess if Dapagliflozin could protect against myocardial ischemia/reperfusion injury and reduce cardiac inflammation	Dapagliflozin Oral gavage (40 mg/kg)	Treatment with Dapagliflozin attenuated infarct size, inhibited inflammation and provided cardioprotection through inhibition of inflammasome assembly
Dapagliflozin, sildenafil and their combination in monocrotaline-induced pulmonary arterial hypertension ***Tang et al.***	2022	Experimental Pulmonary arterial hypertension in rats (Male Sprague-Dawley rats)	To investigate if Dapagliflozin could improve pulmonary vascular remodeling by inhibiting NLRP3 inflammasome activation	Dapagliflozin Oral gavage (1 mg/kg)	Dapagliflozin attenuated right ventricular systolic pressure, pulmonary vascular remodeling and the decreased the NLRP3 activation
Canagliflozin Ameliorates NLRP3 Inflammasome-Mediated Inflammation Through Inhibiting NF-κB Signaling and Upregulating Bif-1 ***Niu et al.***	2022	Experimental Mouse model of autophagy (Swiss male mice)	To evaluate the effects of Canagliflozin on inflammasomes and autophagy	Canagliflozin Oral gavage (20 mg/kg)	Canagliflozin downregulated protein levels of the NLRP3 inflammasome-associated proteins in vivo and in vitro
Empagliflozin Attenuates Obesity-Related Kidney Dysfunction and NLRP3 Inflammasome Activity Through the HO-1-Adiponectin Axis ***Ye et al.***	2022	Experimental Obesity-related kidney disease in obese mice (Male C57BL/6J mice)	Investigate the effects of Empagliflozin on obesity-related kidney disease in obese mice	Empagliflozin Oral gavage (10 mg/kg)	Treatment with Empagliflozin reduced renal injury and NLRP3 inflammasome activation
SGLT2 inhibitor, canagliflozin, ameliorates cardiac inflammation in experimental autoimmune myocarditis ***Long et al.***	2022	Experimental Autoimmune myocarditis in mice (Male BALB/c mice)	Investigate the effects of Canagliflozin over the inflammatory response in an experimental autoimmune myocarditis mouse model	Canagliflozin Oral gavage (30 mg/kg)	Canagliflozin alleviated cardiac inflammation, improved cardiac function and downregulated the expressions of the NLRP3 inflammasome complex components
**In vitro studies**					
The SGLT2 inhibitor Empagliflozin attenuates interleukin-17A-induced human aortic smooth muscle cell proliferation and migration by targeting TRAF3IP2/ROS/NLRP3/Caspase-1-dependent IL-1β and IL-18 secretion ***Sukhanov et al.***	2021	Experimental In vitro – Cell culture (Human aortic SMC and HK-2 the human kidney-2 cells)	Investigate whether Empagliflozin could reduce Oxidative Stress and NLRP3 activity in an in vitro model of aortic smooth muscle cell proliferation	Empagliflozin Concentrations of 0.1 – 5 uM for 15 minutes	Treatment with Empagliflozin attenuated oxidative stress, NLRP3 expression, Caspase-1 activation and IL-1β and IL-18 secretion
Empagliflozin protects diabetic pancreatic tissue from damage by inhibiting the activation of the NLRP3/caspase-1/GSDMD pathway in pancreatic β cells: in vitro and in vivo studies ***Liu et al.***	2021	Experimental In vitro (pancreatic B cells) and In vivo (mouse) (Male BKS-Leprem2Cd479/Gpt mice and male C57BL/6 mice)	Investigate the effects of Empagliflozin over the NLRP3 inflammasome in pancreatic tissues of diabetic mice and in pancreatic B cells	Empagliflozin Concentrations of 50, 100, 200 and 500 nmol/L (in vitro) and oral gavage (10 mg/kg – in vivo)	Empagliflozin reduced the expression levels of NLRP3/Caspase-1/GSDMD in vitro and in vivo, and reduced pathological changes and inflammatory infiltration in pancreatic tissues

In the context of DKD, modulation of the inflammatory response has been observed in different animal models using SGLT2i, which attenuated the progression of kidney disease^80,81,82^. The use of SGLT2i has already demonstrated glycemic benefits and a reduction of pro-inflammatory markers^82^ through inhibition of macrophage activity^83^, suppression of molecular pathways involving PPRs^78,81,84^ and reduction of oxidative stress^85^.

**Figure 1:**
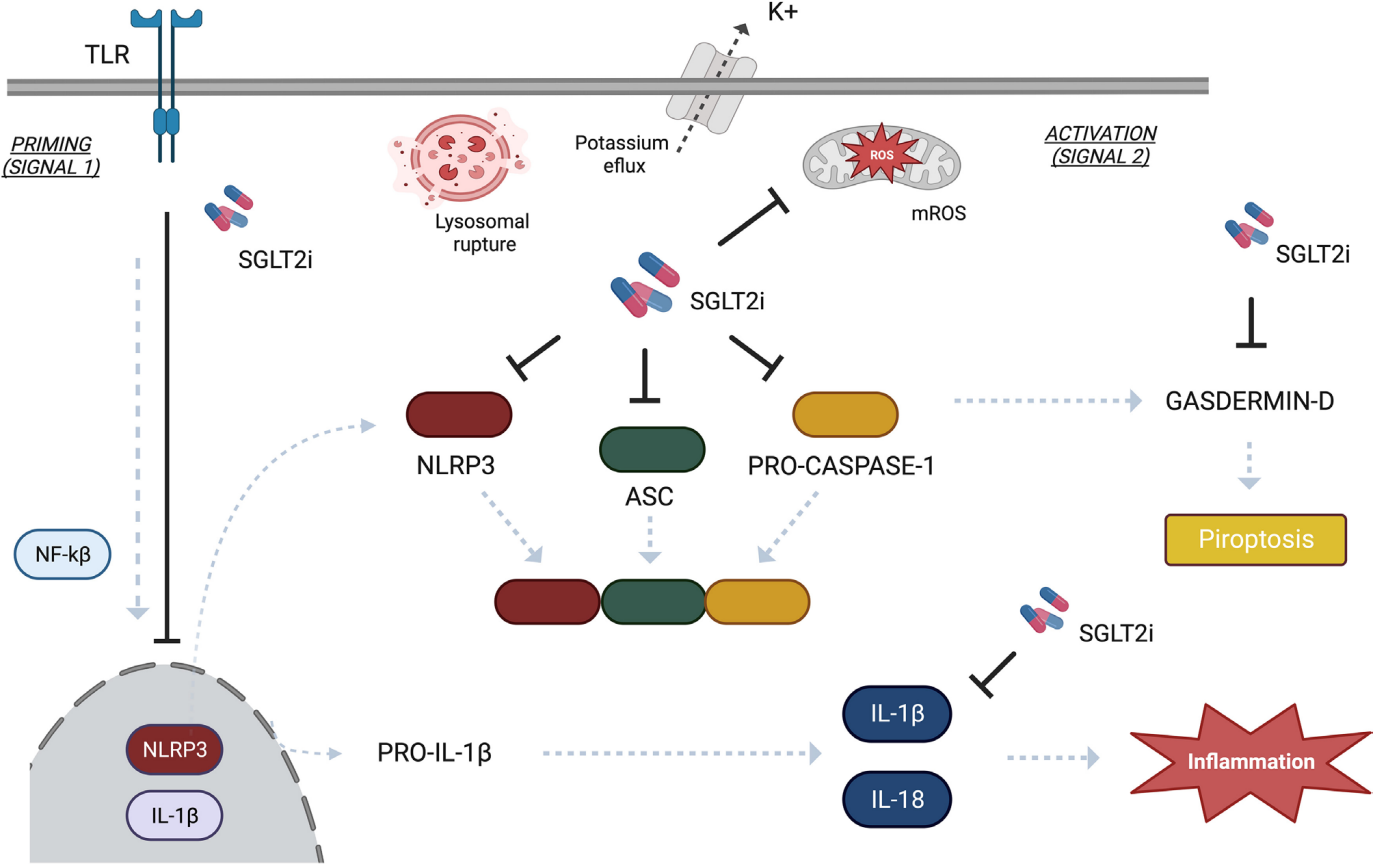
NLRP3 inflammasome activation and the possible mechanisms of action of SGLT2i. The NLRP3 inflammasome activation relies on two key mechanisms. Firstly, NF-kB activation in the nucleus prompts the transcription of pro-IL-1β, pro-IL-18, and NLRP3 enzyme. Subsequently, a secondary signal triggers NLRP3 complex activation through cleavage of pre-activated precursors by stimuli like DAMPs or increased ROS concentration, leading to cytokine release and pyroptosis. SGLT2i potentially inhibits NF-kB, NLRP3, ASC, and Pro-caspase-1 activities, Gasdermin-D activation, and reduces IL-1β levels and mitochondrial reactive oxygen species (mROS).

Concerning the modulation of the NLRP3 complex in the context of DKD, the few studies that have evaluated this relationship have already demonstrated a reduction in the activity of the complex^86^, which has led to an improvement in glycemic levels and a reduction in the progression of kidney disease, with functional and histological advances^58,87,88^. The full activation process of the NLRP3 inflammasome and the possible relationship with SGLT2i is shown in Figure 1.

In 2014, Tahara et al. demonstrated a positive impact on glycemic levels and a reduction of pro-inflammatory markers (IL-6 and TNF-α), chemokines, and oxidative stress with the use of ipragliflozin in a model of T1DM induced by streptozotocin in rats^82^, and these benefits were also replicated in a model of T2DM induced by streptozomicin and a hypercaloric diet in mice^89^.

In 2016, Benetti et al. evaluated for the first time the effects of an SGLT2 inhibitor on the NLRP3 complex in the context of metabolic syndrome in an animal model of diet-induced obesity and insulin resistance^87^. In this study, the authors demonstrated for the first time that empaglifozin treatment was able to improve glycemic levels and pathophysiological changes in metabolic syndrome, in addition to reducing NLRP3 complex activation, with IL-1β inhibition in a dose-dependent manner. Also, the authors found that animals treated with iSGTL2 showed fewer pathological changes in their histology, especially less tubular vacuolation, which is one of the first signs of kidney tubule degeneration.

In 2017, Ye et al. assessed the use of dapagliflozin in the interaction with the NLRP3 complex and the progression of diabetic cardiomyopathy in mice with type 2 diabetes^88^. The findings revealed a reduction in pathological cardiac remodeling, a decrease in glycemic levels in glucose tolerance tests and a decrease in mRNA levels of NALP3, ASC, IL-1β, IL-6, and caspase-1. In 2018, Birnbaum et al. demonstrated that the use of dapagliflozin could attenuate the inflammatory response and thus kidney injury and glomerulosclerosis in diabetic rats by reducing the expression levels of ASC mRNA, caspase-1, IL-6, IL-1β and TNF-α^86^.

## Future Perspectives

Studies involving the influence of SGLT2 inhibitors on the inflammasome complex have opened up new avenues for the treatment of DKD. However, there is still much inconclusive information. Therefore, future perspectives for studies on the influence of SGLT2 inhibitors on the inflammasome complex in DKD should include:

1. Elucidation of the molecular mechanisms involved in the regulation of the inflammasome complex by SGLT2 inhibitors. Although studies have shown that SGLT2 inhibitors reduce NLRP3 inflammasome activation, the precise molecular mechanisms involved are not fully understood.
2. The use of SGLT2 inhibitors in combination with other therapies. Currently available SGLT2 inhibitors have been shown to be effective in the treatment of DM. However, there is still room for improvement in terms of effectiveness and safety. Future research could focus on the clinical and molecular responses of the new SGLT2 inhibitors when combined with physical activity, specific diets, and nutritional supplements, among others.
3. Clinical trials to evaluate the long-term effects of SGLT2 inhibitors on the progression of DKD. Although several studies have shown that SGLT2 inhibitors improve the inflammasome complex and kidney function in people with DKD, the long-term effects and timeframe of these medications in the progression of the disease are still not fully understood.
4. Identification of biomarkers that predict response to SGLT2 inhibitors. Not all people with diabetic kidney disease respond to SGLT2 inhibitors. Biomarkers that predict response to these drugs can help identify individuals most likely to benefit from treatment.

In conclusion, the studies on the influence of SGLT2 inhibitors on the inflammasome complex have increased knowledge of the potential therapeutic effect in the treatment of DKD. Future studies could focus on elucidating the molecular mechanisms involved, evaluating the use of complementary therapies to achieve better efficacy and safety profiles, assessing the long-term effects of these drugs, and identifying biomarkers to predict response to such treatment.

## Data Availability

All datasets generated and analyzed during the current study are available from the corresponding author on reasonable request.
