# Supplementary material for: SGLT2 inhibitors and NLRP3 inflammasome: potential target in diabetic kidney disease
Source: J Bras Nefrol. 2024 Sep 9;46(4):e20230187. doi: 10.1590/2175-8239-JBN-2023-0187en (PMC11539899; doi:10.1590/2175-8239-JBN-2023-0187en)
Supplement: Translation

# Translation: Inibidores de SGLT2 e inflamassoma NLRP3: alvo potencial na doença renal do diabetes

**DOI:** 10.1590/2175-8239-JBN-2023-0187pt

**Authors:** Paulo André Bispo Machado, André Lass, Bruna Isadora Pilger, Raphaella Fornazari, Thyago Proença de Moraes, Ricardo Aurino Pinho

**Affiliations:** 1 Pontifícia Universidade Católica do Paraná, Laboratório de Bioquímica do Exercício em Saúde, Curitiba, PR, Brasil.; 2 Pontificia Universidade Católica do Paraná, Pós-graduação em Ciências da Saúde, Curitiba, PR, Brasil.; 3 Pontifícia Universidade Católica do Paraná, Curitiba, PR, Brasil.

**Keywords:** Nefropatia Diabética, Resposta Inflamatória, Proteína 3 contendo domínio de pirina da família NLR, Inflamassoma, Inibidores do Transportador de Sódio-Glicose 2, Insuficiência Renal Crônica

## Abstract

A doença renal do diabetes (DRD) permanece como a principal causa de doença renal crônica (DRC) mundialmente. A patogênese da DRD é influenciada por mecanismos funcionais, histopatológicos e imunológicos, incluindo a atividade do inflamassoma NLRP3 e estresse oxidativo. Os inibidores do cotransportador de sódio-glicose 2 (iSGLT2) demonstraram benefícios metabólicos e a capacidade de retardar a progressão da DRD em diversos estudos clínicos ao longo dos anos. Estudos recentes sugerem que a atividade antidiabética também se estende à inibição da resposta inflamatória, incluindo modulação do inflamassoma NLRP3, redução de marcadores pró-inflamatórios e redução do estresse oxidativo. Aqui, avaliamos a eficácia dos iSGLT2 no tratamento da DRC e discutimos o papel da resposta inflamatória no desenvolvimento da DRD, incluindo sua relação com o inflamassoma NLRP3 e o estresse oxidativo.

## Resumo Clínico

- A doença renal do diabetes é a principal causa de doença renal crônica
- O inflamassoma NLRP3 é um regulador chave da resposta inflamatória no desenvolvimento de doença renal do diabetes
- Os inibidores do SGLT2 demonstraram capacidade de retardar a doença renal do diabetes em diversos estudos clínicos
- Os inibidores do SGLT2 podem retardar a progressão da doença renal do diabetes ao inibir o inflamassoma NLRP3

## Doença Renal Crônica: Definição e Epidemiologia

A doença renal crônica (DRC) é um problema de saúde pública com impacto global, caracterizada por uma perda progressiva e irreversível da função renal^1^. Nos últimos anos, estimou-se um aumento na incidência de DRC como resultado de alterações demográficas, com a DRC afetando aproximadamente 9% da população mundial^2^. Para sua caracterização, o requisito temporal é fundamental, definida pela presença de dano renal (albuminúria ≥ 30 mg/dia) e/ou uma taxa de filtração glomerular < 60 mL/min/1,73 m^2^ por um período maior ou igual a 3 meses, com perda progressiva, irreversível e geralmente assintomática da função renal^3^.

A DRC apresenta uma etiologia multifatorial e suas principais causas são diabetes mellitus (DM), hipertensão arterial sistêmica (HAS), obesidade e histórico familiar^2,3^. O DM é um distúrbio metabólico intermediário caracterizado por elevações persistentes dos níveis de glicose plasmática. A prevalência de DM aumentou significativamente nos últimos anos, principalmente devido a estilos de vida sedentários, obesidade e aumento da expectativa de vida. Em 1985, cerca de 30 milhões de casos foram registrados em todo o mundo, aumentando para 415 milhões em 2015 e estima-se que haverá 642 milhões de casos até 2040^4^.

O desenvolvimento da doença renal do diabetes (DRD) está relacionado principalmente à duração do diabetes e ao rigor do controle glicêmico, com diversas complicações macrovasculares e microvasculares associadas, incluindo doença arterial obstrutiva periférica, retinopatia diabética e DRD ou nefropatia diabética (ND) – as complicações crônicas mais comuns do DM. Estima-se que aproximadamente 40% dos indivíduos com DM irão desenvolver DRC, que se manifesta como DRD. Isso a torna a principal causa de DRC globalmente^3^ e uma das principais causas de óbito nesses pacientes^5^.

A patogênese da DRD é influenciada por mecanismos funcionais, histopatológicos e imunológicos. Do ponto de vista funcional, a hiperglicemia crônica causa um processo de hiperfiltração glomerular e um aumento na pressão glomerular, que se caracteriza clinicamente por um desequilíbrio no tônus muscular das arteríolas aferente e eferente, resultando em aumento da taxa de filtração glomerular (TFG), hipertensão glomerular e aumento da excreção de albumina na urina para níveis superiores a 30 mg/g^3^. Histologicamente, manifesta-se por glomeruloesclerose nodular e/ou difusa, fibrose tubulointersticial, dilatação mesangial e espessamento da membrana basal, todas alterações consideradas características da DRD^6^.

Com o tempo, o aumento sustentado da TFG associado à hipertensão glomerular leva ao estresse mecânico na barreira de filtração, resultando na perda de função dos néfrons, o que, por sua vez, leva à proteinúria e ao desenvolvimento de DRC^7^. Uma coorte (RIACE) demonstrou que a hiperfiltração glomerular é um fator independente para óbito por qualquer causa em pacientes com DM tipo 2 (DMT2)^8^. Minutolo et al.^9^ mostraram que a presença de albuminúria está associada a um aumento na mortalidade e eventos cardiovasculares em pacientes com DRD em comparação com pacientes com DRC de etiologia não diabética, com o risco diretamente relacionado à gravidade da albuminúria.

## Tratamento Farmacológico

O tratamento farmacológico é um pilar importante no tratamento da DRD, atuando principalmente no controle glicêmico. Nos estágios iniciais tanto do DM tipo 1 (DMT1) quanto do DMT2, as complicações microvasculares e macrovasculares podem ser reduzidas por meio de um controle glicêmico rigoroso^10^.

Os medicamentos para diabetes administrados por via oral podem agir de diversas maneiras, incluindo a redução da produção hepática de glicose (biguanidas/metformina), aumento da secreção de insulina (glinidas e sulfonilureias), redução da absorção intestinal de glicose (inibidores da alfa-glicosidase), aumento da sensibilidade periférica à insulina (tioglitazonas), redução do esvaziamento gástrico (agonistas do GLP-1), aumento da meia-vida do GLP-1 (inibidores da DPP4) e, mais recentemente, estimulação da excreção urinária de glicose (inibidores do SGLT2)^11^.

Os inibidores da enzima conversora de angiotensina II (ECA) ou os bloqueadores dos receptores de aldosterona (BRAs) também são medicamentos essenciais no tratamento da DRD, pois podem reduzir a hiperfiltração e a hipertensão glomerular por meio da vasoconstrição da arteríola aferente e da vasodilatação da arteríola eferente. Estudos clínicos importantes demonstraram que o uso de inibidores da ECA em pacientes com albuminúria reduz a progressão da DRC. Esses medicamentos são responsáveis pelo controle neuro-hormonal dos rins e seu uso permite a otimização da hemodinâmica renal devido ao estado pró-inflamatório presente em pacientes renais^12,13^. Apesar dos benefícios, esses medicamentos não são capazes de eliminar o aumento do risco de mortalidade a longo prazo nesses pacientes^14,15^.

## Inibidores do SGLT2

Os inibidores do cotransportador de sódio-glicose 2 (iSGLT2) são uma nova classe de agentes antidiabéticos orais lançada em 2012, com objetivo de retardar a progressão da doença reduzindo os níveis de glicose no sangue através da urina^16^, demonstrando benefícios metabólicos e capacidade de retardar a progressão da DRD em vários estudos clínicos nos últimos anos.

Os iSGLT2 atuam nos túbulos renais proximais e inibem a reabsorção de glicose independentemente da insulina ou da função das beta pancreáticas. Nesse sentido, os iSGLT2 têm sido alvo de grandes estudos devido ao seu benefício comprovado nos desfechos cardiovasculares e renais^17^, principalmente por proporcionarem melhor controle glicêmico ao regular a hemoglobina glicosilada, glicemia de jejum e glicemia pós-prandial, redução da pressão arterial devido à perda de sódio urinário^18^, e perda de peso^19^.

No estudo DAPA-CKD, o uso de dapagliflozina reduziu significativamente o risco de perda da função renal (estimada pela TFG), progressão da DRC e óbito relacionado à função renal em pacientes com DRC, independentemente do status glicêmico^20^. O estudo também mostrou que pacientes não diabéticos com DRC que receberam dapagliflozina apresentaram melhora da função renal (também estimada pela TFG). Outro estudo de referência na literatura médica é o estudo *EMPA Kidney*, que mostrou que a empagliflozina reduziu o risco de progressão da doença renal ou morte cardiovascular em pacientes com DRC em 28% quando comparado ao placebo^21^.

Os SGLTs são transportadores transmembrana dependentes de ATP que atuam como transportadores de glicose, íons e aminoácidos, sendo os subtipos mais estudados o SGLT1 e o SGLT2^22^. O subtipo SGLT1 é menos específico, pois é encontrado em vários órgãos, como intestino delgado, cérebro, coração e segmentos distais dos túbulos renais proximais (segmento S3) e é responsável pela reabsorção de aproximadamente 10% da glicose filtrada pelo rim^17^. Por estarem localizados em diferentes partes do corpo, os inibidores do subtipo SGLT1 podem causar diversos efeitos adversos, como distúrbios gastrointestinais, o que levou à interrupção dos estudos que inicialmente envolviam esse subtipo^22^. Por outro lado, o subtipo SGLT2 é um transportador específico do tecido renal com baixa afinidade e elevada capacidade de reabsorção de glicose, encontrado na membrana apical das células proximais dos túbulos renais (segmento S1), responsável pela reabsorção de aproximadamente 90% da glicose filtrada pelos rins^16^.

O primeiro medicamento da classe dos iSGLT2 a ser descoberto foi a florizina, uma molécula encontrada nas folhas e nos brotos das macieiras^23^. Porém, devido à sua baixa seletividade para SGLT2 e a diversos efeitos colaterais, principalmente gastrointestinais, a droga deixou de ser utilizada^24^. No entanto, outros medicamentos foram desenvolvidos com base em sua estrutura molecular, aumentando a seletividade para o SGLT2, reduzindo assim os efeitos colaterais e aumentando a biodisponibilidade.

Até o momento, existem aproximadamente 9 moléculas que podem inibir o transportador SGLT2, mas somente 4 foram aprovadas pela *Food and Drug Administration* (FDA) para o tratamento de DM2, a saber: empagliflozina, dapagliflozina, canagliflozina e ertagliflozina^25^. A dapagliflozina foi o primeiro medicamento dessa classe a ser aprovado em 2012, enquanto a empagliflozina foi aprovada em 2014^26^. Na classe, a empagliflozina apresenta maior afinidade pelo SGLT2 em comparação com o SGLT1^27,28,29^.

Nos túbulos proximais, a proporção sódio-glicose é de 1:1 para SGLT2 (segmento S1) e 2:1 para SGLT1 (segmento S3)^30^. A glicose reabsorvida é então transferida para o interstício por difusão facilitada através dos transportadores de glicose (GLUTs) presentes na membrana basolateral da célula^31^.

Estima-se que um adulto saudável possa filtrar aproximadamente 180 g de glicose por dia^32^. Em níveis mais altos, a capacidade do rim de reabsorver a glicose é prejudicada, de modo que níveis elevados de glicose são detectados em exames de urina, causando sintomas de hiperglicemia, como poliúria e polidipsia. Paradoxalmente, essa perda de glicose em indivíduos diabéticos leva à hipertrofia das células dos túbulos renais proximais, onde está localizado o transportador SGLT2, com um aumento de sua expressão na membrana apical, elevando a capacidade de reabsorção de glicose renal por esse transportador, como tentativa de preservar a energia corporal.

Dessa forma, há aumento na reabsorção de glicose e sódio, com redução na disponibilidade de NaCl no lúmen dos túbulos distais, próximos à mácula densa. Esse processo desencadeia o feedback tubuloglomerular, promovendo relaxamento do tônus da arteríola aferente do rim, aumentando o fluxo sanguíneo e a TFG, causando hipertensão glomerular^32^. Esse processo aumenta ainda mais os níveis glicêmicos já previamente elevados, levando ao aumento da secreção de insulina e ao ganho de peso corporal^33^.

Ao bloquear o transportador SGLT2, essa classe de medicamentos promove uma redução na absorção de sódio, promovendo efeitos importantes na dinâmica da filtração renal. O primeiro efeito está relacionado à excreção de sódio na urina, resultando em redução da pressão arterial e do volume circulante efetivo^34^. O segundo efeito está relacionado ao feedback tubuloglomerular, no qual o aumento da presença de sódio na mácula densa gera vasoconstrição da arteríola aferente, reduzindo, dessa forma, a hiperfiltração glomerular^34^.

Estudos experimentais em ratos demonstraram que o uso de iSGLT2 pode reduzir a hiperfiltração em aproximadamente 25% por meio da diminuição da reabsorção de NaCl e glicose nos túbulos proximais, reduzindo assim a pressão intraglomerular (5–8 mmHg)^35^, sendo a redução da pressão intraglomerular um fator importante na redução da albuminúria promovida pelos iSGLT2^35^. Outra hipótese sobre o mecanismo de ação dos iSGLT2 está relacionada às alterações no metabolismo lipídico e ao aumento da produção de corpos cetônicos^36^ bem como às alterações hemodinâmicas^35^.

## Resposta Inflamatória e Estresse Oxidativo Renal

Estudos recentes demonstraram que a resposta inflamatória e o estresse oxidativo são importantes no desenvolvimento e progressão da DRD. Isso se deve principalmente ao sistema imunológico inato, ao aumento de espécies reativas de oxigênio (EROs) e ao aumento de células pró-inflamatórias e citocinas, como interleucina-1ß, interleucina-18, fator de necrose tumoral alfa (TNF-α) e fator nuclear kB (NF-kβ)^37,38,39,40^.

Historicamente, acreditava-se que a DRD era uma doença metabólica caracterizada apenas por hiperglicemia associada a fatores de risco cardiometabólicos, como hipertensão arterial, obesidade e dislipidemia^38^. Apesar disso, diversos estudos têm destacado a complexidade da doença e descrito a importância da imunidade inata, da resposta inflamatória e do estresse oxidativo no seu desenvolvimento e progressão^39,40^.

O complexo inflamassoma NLRP3, um receptor intracitoplasmático encontrado em células do sistema imunológico inato, é um regulador chave desse mecanismo inflamatório. Sabe-se que ele desempenha um papel na fisiopatologia de doenças neurodegenerativas e cardiovasculares, como Alzheimer e insuficiência cardíaca e, recentemente, tem sido estudado em relação a doenças metabólicas, como o DMT2^40^.

Além disso, o estresse oxidativo é um fator crucial na patogênese da DRD. Na DRD, o dano oxidativo aos rins é causado por um aumento na produção de espécies reativas de oxigênio (EROs) e uma diminuição nos mecanismos de defesa antioxidante. Na DRD, a hiperglicemia, dislipidemia e ativação do sistema renina-angiotensina-aldosterona contribuem para o estresse oxidativo^41,42^.

As EROs são geradas por enzimas da cadeia respiratória mitocondrial e podem danificar diretamente os lipídios, proteínas e o DNA, além de ativar múltiplas vias de sinalização que contribuem para inflamação e fibrose. Por exemplo, a hiperglicemia ativa uma via metabólica específica envolvendo diacilglicerol (DAG), proteína quinase C (PKC) e NADPH oxidase, resultando na produção de EROs^42^.

Na DRD, a hiperglicemia crônica aumenta a produção de EROs e induz a apoptose celular, contribuindo para a progressão de complicações diabéticas. Assim, a redução do estresse oxidativo por meio de diversos mecanismos (modificações no estilo de vida, medicamentos e terapias antioxidantes direcionadas) pode prevenir ou retardar a progressão da DRD^43^.

## A Imunidade Inata

A imunidade inata é a primeira linha de defesa do corpo contra agentes infecciosos. É composta principalmente pelas células natural killer, o sistema complemento e o sistema fagocitário. O sistema fagocitário, especialmente representado por macrófagos, é importante para a produção de citocinas pró-inflamatórias, EROs e metaloproteinases, responsáveis pela inflamação local, aterogênese e dano tecidual^44,45,46^.

Diversos estudos já demonstraram que a magnitude de infiltração dessas células no parênquima renal está diretamente relacionada ao grau de disfunção funcional dos rins, sugerindo uma possível relação causal^47,48^. Furuta et al. foram os primeiros a observar esse fenômeno em 1993, ao estudar a infiltração de macrófagos em biópsias de pacientes com DM^49^ e sugeriram que o sistema fagocitário pode estar relacionado ao dano irreversível à estrutura glomerular. Em 2006, Nguyen et al. demonstraram um acúmulo de macrófagos e células imunes nos túbulos renais em tecidos de biópsia de pacientes com DRD, associando a intensidade do acúmulo intersticial ao declínio da função renal, evidenciando a importância dessas células na patogênese da doença^50^.

Ninichuk et al. demonstraram, no ano seguinte, que ao bloquear o CCR-1, uma quimiocina que atrai macrófagos para o tecido renal, foi possível reduzir a fibrose tubular e a inflamação intersticial em um modelo animal de DRD^51^. Em 2017, Klessens et al. usaram um método semelhante para demonstrar o acúmulo de macrófagos nos glomérulos a partir de biópsias de 88 pacientes com doença renal do diabetes e associaram o acúmulo fagocitário como um fator importante na progressão da doença^52^. O aumento na expressão de quimiocinas e moléculas de adesão (CCR-1, CCR-2) já foi observado em biópsias renais de pacientes com doença renal diabética^53,54^. Estudos que avaliaram o uso de antagonistas desses receptores já demonstraram benefícios em modelos animais, resultando na redução da infiltração de macrófagos no parênquima renal e diminuição da excreção de albumina^54^.

## Receptores de Reconhecimento do Sistema Imunológico

As células do sistema imunológico possuem receptores específicos conhecidos como receptores de reconhecimento de padrões (RRPs), responsáveis pela detecção de moléculas estruturais de microrganismos, denominadas de padrões moleculares associados a patógenos (PAMPs), e outras moléculas como toxinas ou fragmentos de DNA e RNA, proteínas intracelulares como HMGB1 (*High-mobility group B1*) e HSPs (*heat shock proteins*), conhecidos como moléculas associadas a danos teciduais (DAMPs)^55^.

No contexto da DRD, o estado hiperglicêmico crônico pode causar lesões endoteliais e celulares que liberam DAMPs e PAMPs no interstício, que são reconhecidos pelos RRPs. Os RRPs, que podem ser encontrados na membrana plasmática ou no citoplasma celular, trabalham juntos para reconhecer sinais de estresse produzidos pelas células durante a infecção ou lesão celular^56^. Quando esses receptores estão presentes na membrana, eles são chamados de *Toll-Like receptors* (TLRs). Quando expressos na superfície das células renais, eles são em grande parte responsáveis por induzir a resposta imune ao reconhecer DAMPs e PAMPs^57^. Esses DAMPS e PAMPS levam à ativação da resposta imune inata^58^, levando à inflamação renal e dano tecidual, principalmente através da ativação da via NF-kB^59^.

Receptores de lectina tipo C (CLRs), receptores semelhantes ao gene induzível por ácido retinoico (RLRs), receptores similares a ausentes de melanoma (ALRs) e receptores similares ao domínio de oligomerização e ligação de nucleotídeos (NOD) (NLRs) são exemplos de RRPs^60,61^. Os NLRs são responsáveis pela segunda linha de defesa do hospedeiro e são subdivididos em subtipos como NLRP1, NLRP3, NAIP e NLRC4^62^. Cada um deles tem a capacidade de ser ativado especificamente por determinados grupos de PAMPs e/ou DAMPs, e sua ativação leva à formação de complexos proteicos citoplasmáticos conhecidos como inflamassomas.

## O Inflamassoma NLRP3

Os NLRs são receptores citoplasmáticos que atuam como a segunda linha de defesa do hospedeiro e possuem três estruturas funcionais: um domínio central de oligomerização (NATCH), um domínio C-terminal rico em repetições de leucina (LRR) e um domínio N-terminal responsável pela transdução de estímulos com os subdomínios CARD (*Caspase activation and recruitment domain*) ou PYD (*pyrin domain*)^63^. Esses subdomínios são responsáveis por desencadear respostas imunes específicas, fazendo com que os NLRs sejam subdivididos em outros subtipos, como NLRP1, NLRP3, NAIP e NLRC4^62^. O subtipo de receptor cujo mecanismo de função é melhor compreendido no contexto da DRD é o NLRP3^64,65^.

No complexo NLRP3, o domínio N-terminal, responsável pela transdução de estímulos, tem um subdomínio PYD. Por outro lado, a enzima pró-caspase-1 possui um subdomínio CARD. Para que ocorra a interação entre a proteína NLRP3 e a enzima pró-caspase-1, é necessária, portanto, uma proteína adaptadora (ASC) com os domínios PYD e CARD^65^. Através de seu domínio PYD, essa proteína se liga ao NLRP3 e, no lado oposto, a presença de seu subdomínio CARD permite o recrutamento da caspase-1 (via interação CARD-CARD). Após essa interação, ocorre a oligomerização do complexo NLRP3 com a autoclivagem da enzima caspase-1 e subsequente ativação de IL-1β e IL-18. Além disso, a morte celular ocorre mediada pela ativação da gasdermina-D (GSDMD), um processo conhecido como piroptose^65,66^.

Essa ativação do inflamassoma NLRP3 requer dois mecanismos complementares. O primeiro mecanismo está relacionado à estimulação de RRPs localizados na membrana celular por meio de PAMPs ou DAMPs, o que desencadeia uma cascata de sinalização intracelular, culminando na ativação e translocação do fator de transcrição NF-kB para o núcleo celular^67^. Quando o NF-kB entra no núcleo, estimula a transcrição de pró-IL-1β, pró-IL-18 e a expressão da enzima NLRP3^64^.

O segundo sinal de ativação consiste na clivagem de precursores previamente ativados pelo NF-kB no núcleo celular^68,69^. Esse processo é desencadeado pela presença de DAMPs ou outros estímulos, como aumento na concentração de EROs, ATP extracelular, efluxo de potássio, cristais de ácido úrico e proteínas nucleares, como a HMGB1^69,70^. Essas moléculas são capazes de ativar e oligomerizar o complexo NLRP3, envolvendo o recrutamento e a maturação das enzimas caspases, que, ao realizarem um processo de autoclivagem, ativam IL-1β e IL-18, as quais são liberadas no ambiente extracelular por meio de canais criados na membrana citoplasmática^71,72^ para exercer sua ação pró-inflamatória. Além disso, a GSDMD é clivada e seu subdomínio N-terminal é liberado, promovendo a formação de poros na membrana plasmática. Esse processo, conhecido como piroptose, é uma forma de morte celular programada que gera uma intensa resposta inflamatória ao permitir a osmólise celular, lise do DNA e liberação de componentes celulares e mediadores inflamatórios, como IL-1β e IL-18^67,68, 69^.

## O Papel do Complexo NLRP3 na Doença Renal Diabética

Apesar de serem mais comumente encontradas em células do sistema imunológico, relatos prévios já demonstraram que as moléculas constituintes dos inflamassomas estão também presentes em outras células do corpo, a exemplo de podócitos e células mesangiais no sistema renal^72,73^. O subtipo NLRP3 é o mais comum nos rins, estando a ativação desse complexo envolvida no desenvolvimento e progressão da DRD^72,74^.

A descoberta do complexo NLRP3 como componente inflamatório envolvido na patogênese da DRD levou ao desenvolvimento de terapias destinadas a inibir sua atividade com objetivo de atenuar a injúria renal^73,74,75^. No contexto da DRD, demonstrou-se que a modulação do complexo NLRP3 foi capaz de prevenir a inflamação e retardar a progressão da fibrose^67^. Por exemplo, o *knockout* da enzima NLRP3 promoveu redução da resposta inflamatória e do grau de fibrose renal em camundongos diabéticos^75^ e o *knockout* da enzima ASC foi capaz de reduzir a proteinúria e o dano glomerular em camundongos submetidos a uma dieta rica em gordura^76^. Assim, a atenuação da resposta inflamatória por meio da modulação do complexo NLRP3 tornou-se uma nova estratégia terapêutica no contexto das doenças renais, especialmente aquelas relacionadas ao DM, com o objetivo de reduzir a progressão da doença ao diminuir a resposta inflamatória mediada pelo complexo NLRP3.

Um dos estudos mais importantes envolvendo a relação entre o complexo NLRP3 e a DRD foi realizado em 2015 por Shahzad et al. O estudo revelou que o *knockout* da enzima NLPR3 ou da caspase-1 em ratos diabéticos foi capaz de reduzir a albuminúria e o acúmulo de matriz extracelular nesses animais, porém sem alterar os níveis glicêmicos ou o peso corporal^66^. O mesmo estudo também mostrou que os níveis circulantes de IL-1β e IL-18 e a expressão renal de NALP3 estavam elevados em ratos com DRD, sendo que esse aumento precedia o processo de albuminúria e expansão mesangial, o que implica que a ativação do NLRP3 pode ser um gatilho para o desenvolvimento da DRD. Além disso, a inibição farmacológica do receptor de IL-1 foi capaz de reduzir a albuminúria e a expansão mesangial em ratos diabéticos.

Wu et al. demonstraram em 2018 que o *knockout* da enzima NLRP3 em ratos diabéticos também foi capaz de proteger contra a progressão da doença renal diabética. Isso resultou em uma melhora no *cleareance* de creatinina e na relação albumina/creatinina urinária, bem como melhora das consequências histopatológicas, como hipertrofia glomerular e fibrose mesangial e intersticial, em comparação com o grupo controle^75^.

Na mesma linha, diversos medicamentos da classe dos antidiabéticos orais demonstraram potencial para inibir a resposta inflamatória e modular a via do complexo NLRP3 em pacientes diabéticos e em modelos animais. Os exemplos incluem sulfonilureias, biguanidas, glitazonas, agonistas do receptor do peptídeo 1 semelhante ao glucagon (RA-GLP-1) (albiglutida, dulaglutida e exenatida), inibidores da DPP4 e, mais recentemente, inibidores do SGTL2^77,78^.

A exposição glicêmica crônica das células dos túbulos renais promove alterações no metabolismo celular, incluindo aumento da expressão de citocinas pró-inflamatórias, fatores de crescimento e pró-fibróticos e espécies reativas de oxigênio. Assim, a inibição da reabsorção de glicose pelas células do túbulo proximal pode ser um mecanismo de ação pelo qual o iSGLT2 reduz esses efeitos pró-inflamatórios^79^.

## Inibidores do SGLT2 e Resposta Inflamatória

Estudos recentes demonstraram que os inibidores do SGLT2 podem inibir a ativação do inflamassoma NLRP3 em diversos modelos animais, incluindo obesidade, lesão pulmonar, infarto do miocárdio, DRD, depressão e aterosclerose. Acredita-se que essa inibição ocorra por meio de diversos mecanismos, incluindo a redução da captação de glicose e do estresse oxidativo, além da modulação do microbioma intestinal. Os principais artigos que investigaram a possível relação entre os iSGLT2 e o complexo NLRP3 e seus achados são apresentados na Tabela 1.

**Tabela 1:** Estudos avaliando a possível relação entre ISGLT2 e o complexo NLRP3

Título/Autor	Ano	Desenho/Modelo do estudo	Objetivo do estudo	Droga/Método de administração	Resultados do inflamassoma
**Estudos in vivo**					
Empagliflozin Protects against Diet-Induced NLRP-3 Inflammasome Activation and Lipid Accumulation ***Benetti et al.***	2016	Experimental Modelo murino de obesidade induzida por dieta (Ratos C57BL/6J machos)	Avaliar a capacidade da Empagliflozina de afetar o peso corporal e a ativação do inflamassoma NLRP3	Empagliflozina Misturado na dieta (1 mg/kg, 3 mg/kg ou 10 mg/kg)	Redução da ativação da Caspase-1 e na produção de IL-1β em camundongos tratados com Empagliflozina (3 mg/kg e 10 mg/kg)
The SGLT-2 Inhibitor Dapagliflozin Has a Therapeutic Effect on Atherosclerosis in Diabetic ApoE^-/-^Mice ***Leng et al.***	2016	Experimental Aterosclerose diabética em ratos diabéticos (Ratos ApoE-/- e C57BL/6J machos)	Explorar a eficácia da Dapagliflozina na aterosclerose e a influência na via ERO-NLPR3-Caspase	Dapagliflozina Gavagem oral (1 mg/kg)	Os níveis séricos de NLRP3, IL-1β e IL-18 foram reduzidos após 12 semanas de tratamento com Dapagliflozina
SGLT-2 Inhibition with Dapagliflozin Reduces the Activation of the Nlrp3/ASC Inflammasome and Attenuates the Development of Diabetic Cardiomyopathy in Mice with Type 2 Diabetes. Further Augmentation of the Effects with Saxagliptin, a DPP4 Inhibitor ***Ye et al.***	2017	Experimental Cardiomiopatia diabética em ratos (Ratos diabéticos tipo 2 (BTBR ob/ob) e do tipo selvagem (WT))	Avaliar se a Dapagliflozina pode atenuar a disfunção miocárdica e a ativação do NLRP3 em ratos diabéticos	Dapagliflozina Misturado na dieta (1 mg/kg)	A Dapagliflozina atenuou a ativação do inflamassoma NLRP3 (níveis mais baixos de RNAm de ASC, NALP3, IL-1β e Caspase-1)
Combined SGLT2 and DPP4 Inhibition Reduces the Activation of the Nlrp3/ASC Inflammasome and Attenuates the Development of Diabetic Nephropathy in Mice with Type 2 Diabetes ***Birnbaum et al.***	2018	Experimental Nefropatia Diabética em ratos (Ratos BTBR ob/ob e WT machos)	Foi avaliado se a Dapagliflozina atenua a ativação do NLRP3 e a progressão da ND em ratos	Dapagliflozina Misturado na dieta (1 mg/kg)	A Dapagliflozina atenuou a ativação do inflamassoma NLRP3 após 8 semanas
The SGLT2 inhibitor dapagliflozin attenuates the activity of ROS-NLRP3 inflammasome axis in steatohepatitis with diabetes mellitus ***Leng et al.***	2019	Experimental Esteatohepatite em ratos diabéticos (Ratos ApoE-/-)	Avaliar os efeitos da Dapagliflozina na lesão hepática e a influência no estresse oxidativo e na atividade do NLRP3	Dapagliflozina Gavagem oral (1 mg/kg)	A Dapagliflozina reduziu a atividade do inflamassoma NLRP3 no fígado (NLRP3, Caspase-1, IL-1β e IL-18)
Empagliflozin Blunts Worsening Cardiac Dysfunction Associated with Reduced NLRP3 (Nucleotide-Binding Domain-Like Receptor Protein 3) Inflammasome Activation in Heart Failure ***Byrne et al.***	2020	Experimental Modelo de insuficiência cardíaca em ratos (Ratos C57BL/6J machos e ratos Dahl machos sensíveis ao sal)	Avaliar os efeitos da Empagliflozina no inflamassoma NLRP3 em um modelo de insuficiência cardíaca em camundongos	Empagliflozina Gavagem oral (10 mg/kg)	A Empagliflozina reduziu os níveis de IL-1β e IL-18, a infiltração de macrófagos e os níveis de transcrição de NLRP3, NF-kB e Caspae-1
Dapagliflozin and Ticagrelor Have Additive Effects on the Attenuation of the Activation of the NLRP3 Inflammasome and the Progression of Diabetic Cardiomyopathy: an AMPK-mTOR Interplay ***Chen et al.***	2020	Experimental Cardiomiopatia diabética em ratos diabéticos (Ratos BTBR e WT)	Avaliar se a Dapagliflozina (e o Ticagrelor) atenuaria a progressão da cardiomiopatia diabética no DMT2 em camundongos	Dapagliflozina Misturado na água potável (1,5 mg/kg)	A Dapagliflozina reduziu os níveis de RNAm de ASC, Caspase-1 e NLRP3 após 12 semanas
SGLT2 inhibition modulates NLRP3 inflammasome activity via ketones and insulin in diabetes with cardiovascular disease ***Kim et al.***	2020	Ensaio Clínico Randomizado Adultos (20–79 anos) com DMT2 (Humanos)	Investigar os efeitos da Empagliflozina sobre o inflamassoma NLRP3 em pacientes com DMT2 e alto risco de doença cardiovascular	Empagliflozina Oral – 10 mg ou 25 mg	A Empagliflozina reduziu os níveis de IL-1B em macrófagos e os níveis de RNAm de IL-1β, TNF-A e NLPR3 após 30 dias
Dapagliflozin, an SGLT2 inhibitor, ameliorates acetic acid-induced colitis in rats by targeting NFκB/AMPK/NLRP3 axis ***El Rous et al.***	2021	Experimental Colite Ulcerativa em ratos (Ratos Sprague-Dawley machos)	Investigar os efeitos da Dapagliflozina em um modelo de Colite Ulcerativa em ratos e a possível modulação do complexo NLRP3/NF-kB	Dapagliflozina Gavagem oral (5 mg/kg ou 10 mg/kg)	Sete dias de tratamento com Dapagliflozina (10 mg/kg) foram capazes de suprimir a sinalização de NF-kB, bem como a atividade de NLRP3/Caspase-1
Ticagrelor and Dapagliflozin Have Additive Effects in Ameliorating Diabetic Nephropathy in Mice with Type-2 Diabetes Mellitus ***Birnbaum et al.***	2021	Experimental Nefropatia Diabética em ratos (Ratos BTBR ob/ob machos)	Foi avaliado se a Dapagliflozina (ou sua associação com o Ticagrelor) atenua a ativação do NLRP3 e a progressão da ND em camundongos	Dapagliflozina Misturado na água potável (1,5 mg/kg)	A Dapagliflozina atenuou a ativação do inflamassoma NLRP3 após 12 semanas
The SGLT-2 inhibitor empagliflozin improves myocardial strain, reduces cardiac fibrosis and pro-inflammatory cytokines in non-diabetic mice treated with doxorubicin ***Quagliariello et al.***	2021	Experimental In vitro (cardiomiócito) e in vivo (ratos) (Ratos C57BL/6 fêmeas)	Avaliar os efeitos da Empagliflozina (e sua associação com a Doxorrubicina) sobre o inflamassoma NLRP3 em culturas de células de cardiomiócitos e em um modelo in vivo	Empagliflozina Concentrações de 50, 100 e 500 nM (in vitro) e gavagem oral (10 mg/kg – in vivo)	A Empagliflozina reduziu a atividade do NF-kB (de forma dose-dependente) e também a atividade do complexo NLRP3
Crosstalk Among NLRP3 Inflammasome, ET_B_R Signaling, and miRNAs in Stress-Induced Depression-Like Behavior: a Modulatory Role for SGLT2 Inhibitors ***Muhammad et al.***	2021	Experimental Modelo de depressão em ratos (Ratos Wistar machos)	Avaliar os efeitos da Dapagliflozina como modalidade terapêutica em um modelo animal de depressão e a possível relação com o Inflamassoma NLRP3	Dapagliflozina oral (1 mg/kg)	A dapagliflozina foi capaz de suprimir os níveis de IL-1β e IL-18, bem como a atividade do complexo NLRP3 após 4 semanas
SGLT2 inhibitor counteracts NLRP3 inflammasome via tubular metabolite itaconate in fibrosis kidney ***Ke et al.***	2022	Experimental Fibrose renal em modelo de isquemia/reperfusão em ratos (Ratos C57BL/6J machos)	Avaliar se a Dapagliflozina reduziria a fibrose renal por meio da inibição do complexo NLRP3 em um modelo de isquemia/reperfusão	Dapagliflozina Gavagem oral (1 mg/kg)	A dapagliflozina reverteu as alterações no metabolismo energético e na inflamação e preveniu a ativação do NLRP3
The dynamic interplay between AMPK/NFκB signaling and NLRP3 is a new therapeutic target in inflammation: Emerging role of dapagliflozin in overcoming lipopolysaccharide-mediated lung injury ***El-Fattah et al.***	2022	Experimental Lesão pulmonar aguda em ratos (Ratos Sprague-Dawley machos)	Avaliou-se o papel da Dapagliflozina em um modelo de lesão pulmonar induzida por lipopolissacarídeo em ratos	Dapagliflozina Gavagem oral (5 mg/kg ou 10 mg/kg)	A Dapagliflozina mitigou a lesão pulmonar, reduziu o estresse oxidativo e inibiu a atividade do inflamassoma NLRP3 e da caspase-1 através da regulação da AMPK/NFkB
Dapagliflozin Alleviates Renal Fibrosis by Inhibiting RIP1-RIP3-MLKL-Mediated Necroinflammation in Unilateral Ureteral Obstruction ***Xuan et al.***	2022	Experimental Fibrose renal em modelo de obstrução unilateral do ureter (Ratos Sprague-Dawley machos)	Investigar se a Dapagliflozina pode proporcionar proteção renal contra a fibrose renal por meio de efeitos anti-inflamatórios em um modelo de obstrução ureteral em ratos	Dapagliflozina Gavagem oral (10 mg/kg)	A dapagliflozina atenuou a fibrose renal, a expressão de parâmetros pró-inflamatórios (IL-1β, IL-18 e NLRP3) e o estresse oxidativo
Sodium-Glucose Co-transporter-2 Inhibitor of Dapagliflozin Attenuates Myocardial Ischemia/Reperfusion Injury by Limiting NLRP3 Inflammasome Activation and Modulating Autophagy ***Yu et al.***	2022	Experimental Modelo de lesão de isquemia/reperfusão miocárdica (Ratos C57BL/6J machos)	Avaliar se a Dapagliflozina pode proteger contra lesão de isquemia/reperfusão do miocárdio e reduzir a inflamação cardíaca	Dapagliflozina Gavagem oral (40 mg/kg)	O tratamento com Dapagliflozina atenuou o tamanho do infarto, inibiu a inflamação e proporcionou cardioproteção através da inibição da montagem do inflamassoma
Dapagliflozin, sildenafil and their combination in monocrotaline-induced pulmonary arterial hypertension ***Tang et al.***	2022	Experimental Hipertensão arterial pulmonar em ratos (Ratos Sprague-Dawley machos)	Investigar se a Dapagliflozina pode melhorar a remodelação vascular pulmonar pela inibição da ativação do inflamassoma NLRP3	Dapagliflozina Gavagem oral (1 mg/kg)	A Dapagliflozina atenuou a pressão sistólica do ventrículo direito, a remodelação vascular pulmonar e reduziu a ativação do NLRP3
Canagliflozin Ameliorates NLRP3 Inflammasome-Mediated Inflammation Through Inhibiting NF-κB Signaling and Upregulating Bif-1 ***Niu et al.***	2022	Experimental Modelo animal de autofagia (Ratos suíços machos)	Avaliar os efeitos da Canagliflozina em inflamassomas e autofagia	Canagliflozina Gavagem oral (20 mg/kg)	A Canagliflozina reduziu os níveis proteicos das proteínas associadas ao inflamassoma NLRP3 in vivo e in vitro
Empagliflozin Attenuates Obesity-Related Kidney Dysfunction and NLRP3 Inflammasome Activity Through the HO-1-Adiponectin Axis ***Ye et al.***	2022	Experimental Doença renal relacionada à obesidade em ratos obesos (Ratos C57BL/6J machos)	Investigar os efeitos da Empagliflozina na doença renal relacionada à obesidade em camundongos obesos	Empagliflozina Gavagem oral (10 mg/kg)	O tratamento com Empagliflozina reduziu a injúria renal e a ativação do inflamassoma NLRP3
SGLT2 inhibitor, canagliflozin, ameliorates cardiac inflammation in experimental autoimmune myocarditis ***Long et al.***	2022	Experimental Miocardite autoimune em ratos (Ratos machos BALB/c)	Investigar os efeitos da Canagliflozina sobre a resposta inflamatória em um modelo experimental de miocardite autoimune em camundongos	Canagliflozina Gavagem oral (30 mg/kg)	A Canagliflozina aliviou a inflamação cardíaca, melhorou a função cardíaca e reduziu a expressão dos componentes do complexo inflamassoma NLRP3
**Estudos in vitro**					
The SGLT2 inhibitor Empagliflozin attenuates interleukin-17A-induced human aortic smooth muscle cell proliferation and migration by targeting TRAF3IP2/ROS/NLRP3/Caspase-1-dependent IL-1β and IL-18 secretion ***Sukhanov et al.***	2021	Experimental In vitro – Cultura celular (Camada da aorta e células renais humanas HK-2)	Investigar se a Empagliflozina pode reduzir o Estresse Oxidativo e a atividade do NLRP3 em um modelo in vitro de proliferação de células musculares lisas da aorta	Empagliflozina Concentrações de 0,1 – 5 uM por 15 minutos	O tratamento com Empagliflozina atenuou o estresse oxidativo, a expressão do NLRP3, a ativação da Caspase-1 e a secreção de IL-1β e IL-18
Empagliflozin protects diabetic pancreatic tissue from damage by inhibiting the activation of the NLRP3/caspase-1/GSDMD pathway in pancreatic β cells: in vitro and in vivo studies ***Liu et al.***	2021	Experimental In vitro (células B pancreáticas) e in vivo (ratos) (Ratos machos BKS-Leprem2Cd479/Gpt e ratos machos C57BL/6)	Investigar os efeitos da Empagliflozina sobre o inflamassoma NLRP3 em tecidos pancreáticos de camundongos diabéticos e em células B pancreáticas	Empagliflozina Concentrações de 50, 100, 200 e 500 nmol/L (in vitro) e gavagem oral (10 mg/kg – in vivo)	A empagliflozina reduziu os níveis de expressão de NLRP3/Caspase-1/GSDMD in vitro e in vivo, e reduziu as alterações patológicas e o infiltrado inflamatório em tecidos pancreáticos

No contexto da DRD, a modulação da resposta inflamatória foi observada em diferentes modelos animais usando iSGLT2, de forma a atenuar a progressão da doença renal^80,81,82^. O uso de iSGLT2 já demonstrou benefícios glicêmicos e uma redução de marcadores pró-inflamatórios^82^ através da inibição da atividade de macrófagos^83^, supressão de vias moleculares envolvendo RRPs^78,81,84^ e redução do estresse oxidativo^85^.

Quanto à modulação do complexo NLRP3 no contexto da DRD, os poucos estudos que avaliaram essa relação já demonstraram uma redução na atividade do complexo^86^, promovendo melhora nos níveis glicêmicos e a uma redução na progressão da doença renal, com avanços funcionais e histológicos^58,87,88^. O processo completo de ativação do inflamassoma NLRP3 e a possível relação com os iSGLT2 são demonstrados na Figura 1.

**Figura 1:** Ativação do inflamassoma NLRP3 e os possíveis mecanismos de ação dos iSGLT2. A ativação do inflamassoma NLRP3 depende de dois mecanismos principais. Primeiramente, a ativação do NF-kB no núcleo estimula a transcrição das enzimas pró-IL-1β, pró-IL-18 e NLRP3. Posteriormente, um sinal secundário desencadeia a ativação do complexo NLRP3 por meio da clivagem de precursores pré-ativados por estímulos como DAMPs ou aumento da concentração de EROs, levando à liberação de citocinas e piroptose. Os iSGLT2 inibem potencialmente as atividades de NF-kB, NLRP3, ASC e Pro-caspase-1, a ativação de Gasdermin-D e reduzem os níveis de IL-1β e as espécies reativas de oxigênio mitocondriais (EROm).

Em 2014, Tahara et al. demonstraram redução nos níveis glicêmicos e de marcadores pró-inflamatórios (IL-6 e TNF-α), quimiocinas e estresse oxidativo com o uso de ipragliflozina em um modelo de DMT1 induzido por estreptozotocina em ratos^82^, sendo esses benefícios também replicados em um modelo de DMT2 induzido por estreptozotocina e dieta hipercalórica em camundongos^89^.

Em 2016, Benetti et al. avaliaram pela primeira vez os efeitos de um inibidor do SGLT2 no complexo NLRP3 no contexto de síndrome metabólica em um modelo animal de obesidade induzida por dieta e resistência à insulina^87^. Nesse estudo, os autores demonstraram, pela primeira vez, que o tratamento com empaglifozina foi capaz de melhorar os níveis glicêmicos e as alterações fisiopatológicas na síndrome metabólica, além de reduzir a ativação do complexo NLRP3, com inibição da IL-1β de maneira dose-dependente. Além disso, os autores constataram que os animais tratados com iSGTL2 apresentaram menos alterações patológicas na histologia, especialmente menos vacuolização tubular, um dos primeiros sinais de degeneração dos túbulos renais.

Em 2017, Ye et al. avaliaram o uso da dapagliflozina na interação com o complexo NLRP3 e a progressão de cardiomiopatia diabética em camundongos com diabetes tipo 2^88^. Os achados revelaram redução na remodelação cardíaca patológica, uma diminuição nos níveis glicêmicos em testes de tolerância à glicose e uma diminuição nos níveis de RNAm de NALP3, ASC, IL-1β, IL-6 e caspase-1. Em 2018, Birnbaum et al. demonstraram que o uso de dapagliflozina foi capaz de atenuar a resposta inflamatória, a injúria renal e a glomeruloesclerose em ratos diabéticos, reduzindo os níveis de expressão de RNAm de ASC, caspase-1, IL-6, IL-1β e TNF-α^86^.

## Perspectivas Futuras

Estudos envolvendo a influência dos inibidores do SGLT2 sobre o complexo do inflamassoma abriram novos caminhos para o tratamento da DRD. Entretanto, ainda restam informações inconclusivas. Portanto, perspectivas futuras para estudos sobre a influência dos inibidores do SGLT2 sobre o complexo inflamassoma na DRD devem incluir:

1. Elucidação dos mecanismos moleculares envolvidos na regulação do complexo inflamassoma pelos inibidores do SGLT2. Embora estudos tenham demonstrado que os inibidores do SGLT2 reduzem a ativação do inflamassoma NLRP3, os mecanismos moleculares exatos envolvidos não são totalmente compreendidos.
2. O uso de inibidores do SGLT2 em combinação com outras terapias. Os inibidores do SGLT2 atualmente disponíveis demonstraram ser eficazes no tratamento do DM. No entanto, ainda há espaço para melhorias em termos de eficácia e segurança. Pesquisas futuras poderiam se concentrar nas respostas clínicas e moleculares dos novos inibidores do SGLT2 quando combinados com atividade física, dietas específicas e suplementos nutricionais, entre outros.
3. Ensaios clínicos para avaliar os efeitos de longo prazo dos inibidores do SGLT2 na progressão da DRD. Embora diversos estudos tenham demonstrado que os inibidores do SGLT2 melhoram o complexo inflamassoma e a função renal em pessoas com DRD, os efeitos de longo prazo desses medicamentos na progressão da doença ainda não são totalmente compreendidos.
4. Identificação de biomarcadores que predizem a resposta aos inibidores do SGLT2. Nem todas as pessoas com doença renal diabética respondem aos inibidores do SGLT2. Os biomarcadores que preveem a resposta a esses medicamentos podem ajudar a identificar os indivíduos com maior probabilidade de se beneficiarem do tratamento.

Em conclusão, os estudos sobre a influência dos inibidores do SGLT2 no complexo inflamassoma aumentaram o conhecimento sobre o potencial efeito terapêutico no tratamento da DRD. Estudos futuros podem se concentrar em elucidar os mecanismos moleculares envolvidos, avaliar o uso de terapias complementares para obter melhores perfis de eficácia e segurança, avaliar os efeitos de longo prazo desses medicamentos e identificar biomarcadores para prever a resposta a esse tratamento.

## Data Availability

Todos os conjuntos de dados gerados e analisados durante o estudo atual estão disponíveis através do autor correspondente, mediante solicitação adequada.
